# Lipopolysaccharide from *Rhodobacter sphaeroides* Attenuates Microglia-Mediated Inflammation and Phagocytosis and Directs Regulatory T Cell Response

**DOI:** 10.1155/2015/361326

**Published:** 2015-09-17

**Authors:** Sagar Gaikwad, Reena Agrawal-Rajput

**Affiliations:** Laboratory of Immunology, School of Biological Sciences and Biotechnology, Indian Institute of Advanced Research, Gandhinagar, Gujarat 382 007, India

## Abstract

Microglia activation and neuroinflammation are key events during the progression of neurodegenerative disorders. Microglia exhibits toll-like receptors (TLRs), with predominant expression of TLR4, inducing aberrant neuroinflammation and exacerbated neurotoxicity. Studies suggest that microglia initiate infiltration of T cells into the brain that critically influence disease conditions. We report that LPS-Rs, through TLR4 antagonism, significantly inhibit TLR4 mediated inflammatory molecules like IL-1*β*, IL-6, TNF-*α*, COX-2, iNOS, and NO. LPS-Rs regulates JNK/p38 MAPKs and p65-NF-*κ*B signaling pathways, which we report as indispensible for LPS induced neuroinflammation. LPS-Rs mitigates microglial phagocytic activity and we are first to report regulatory role of LPS-Rs which blocked microglia mediated inflammation and apoptotic cell death. LPS-Rs significantly inhibits expression of costimulatory molecules CD80, CD86, and CD40. Chemokine receptor, CCR5, and T cell recruitment chemokines, MIP-1*α* and CCL5, were negatively regulated by LPS-Rs. Furthermore, LPS-Rs significantly inhibited lymphocyte proliferation with skewed regulatory T (Treg) cell response as evidenced by increased FOXP3, IL-10, and TGF-*β*. Additionally, LPS-Rs serves to induce coordinated immunosuppressive response and confer tolerogenic potential to activated microglia extending neurosupportive microenvironment. TLR4 antagonism can be a strategy providing neuroprotection through regulation of microglia as well as the T cells.

## 1. Introduction

Microglia-mediated neuroinflammation and T cell infiltration constitute major hallmarks for neurodegeneration [1], yet the molecular and cellular crosstalk between microglia and T cells is poorly understood. Lipopolysaccharide (LPS) from* Rhodobacter sphaeroides* (LPS-Rs) is a potent TLR4 antagonist in both human and murine cells and prevents TLR4 mediated inflammation [2]. LPS-Rs is a penta-acylated lipid A and utilizes two distinct mechanisms to block LPS/TLR4 signaling. The mechanism involves direct competition between underacylated lipid A and hexa-acylated lipid A for binding on MD-2, whereas another mechanism implies the ability of penta-acylated lipid A:MD-2 complexes to inhibit hexa-acylated endotoxin:MD-2 complexes and TLR4 functions [2–5]. Inhibition of TLR4 signaling is possible by the antagonist. A critical question is asked whether LPS-Rs can be employed to fine-tune the major hallmarks of microglia-mediated inflammation, phagocytosis, and T cell activation.

In resting state, microglia performs maintenance and immune surveillance but activation either by injury or immune stimuli triggers neuroinflammation resulting in neurotoxicity [6]. Microglia expressed toll-like receptors (TLRs) trigger or resolve inflammation and injury and TLR4 is reported to exacerbate microglial activation, neuroinflammation, and lymphocyte infiltration resulting in neurodegeneration [7, 8]. TLR4 functions as receptor for bacterial lipopolysaccharide (LPS) and other endogenous molecules like HSP 70, HSP60, amyloid beta, *α*-synuclein, and so forth [9]. In the study, LPS is taken as an exemplary ligand for TLR4 to elucidate immune regulation at molecular and cellular levels.

TLR4 initiates intracellular signaling that regulates gene expression through phosphorylation of MAPKs and NF-*κ*B. Involvement of the receptor is known in Alzheimer's disease (AD) and Parkinson's disease (PD) [10, 11] and deficiency protects mice against neurodegeneration following injury [12, 13]. Mutation in the receptor decreases microglial activation and preserves cognitive functions [14].

Receptor ligation on antigen presenting cells including microglia would present the processed antigen to the cells of adaptive immune response including the T cells. The microenvironment generated during the course of antigen presentation and inflammation dictates the outcome of  T cell response. The event leads to the priming and recruitment of  T cells through a series of coordinated events. The infiltrating T cells can critically influence the outcome of neurodegeneration that is either resolving it and/or exacerbating it through microglia activation and/or suppression. The inflammatory subset of  T cells including Th1 and Th17 strongly contributes to chronic neuroinflammation, perpetuating neurodegenerative processes, whereas the immunosuppressive Tregs decrease inflammatory functions with neurosupportive microenvironment [15]. Realizing the fact that T cell response can govern the fate of neurodegeneration progression or resolution, in this regard, the fate of  T cell subset differentiation by LPS-Rs was explicated in detail.

BV2 microglia is used as an* in vitro* model to dissect molecular mechanism of LPS-Rs mediated TLR4 antagonism [16]. LPS-Rs has been reported to be nontoxic in rodents [4, 17]. The study gains insight into the mechanisms of LPS-Rs mediated regulation of signaling pathways, prevention of neuroinflammation, and subsequent decrease of neuronal loss following aberrant apoptosis and phagocytosis. Importantly, our study provides novel insights into LPS-Rs mediated Treg generation.

## 2. Materials and Methods

### 2.1. Cell Culture and Treatments

Mouse microglial BV2 cell line kind gift from Dr. Anirban Basu, NBRC, India and mouse neuro2a cell line obtained from National Centre for Cell Science (NCCS), Pune, India were used. The cell lines were maintained in DMEM, 10% heat inactivated fetal bovine serum (FBS), and supplements under appropriate conditions.

For the experiments, cells were washed twice and pretreated with ultrapure LPS-Rs (Invivogen), SB202190-p38 MAPK inhibitor (10 *µ*M), PD184352-ERK1/2 inhibitor (5 *µ*M), SP600125-JNK inhibitor (10 *µ*M), and Curcumin-NF-*κ*B signaling inhibitor (10 *µ*M) (all from Sigma-Aldrich) in 1% FBS containing DMEM for 2 hrs followed by LPS-TLR4 ligand (1 *µ*g/mL) (Sigma-Aldrich). All the reagents were obtained from Life Technologies, USA, unless otherwise mentioned.

### 2.2. Semiquantitative RT-PCR

Total RNA was extracted and quantified and 1 *μ*g of total RNA was used for cDNA synthesis. The cDNA for indicated genes were amplified using gene specific primers (see supplementary Table 1 in Supplementary Material available online at http://dx.doi.org/10.1155/2015/361326) under following conditions: 95°C for 5 min, 95°C for 30 seconds, annealing at 55–60°C for 45 seconds, and 72°C for 1 minute for a total of 20–30 cycles (for different genes) followed by final extension at 72°C for 10 minute and each sample was amplified for *β*-actin to ensure equal cDNA input.

### 2.3. Western Blotting

Expression and phosphorylation of the proteins were assayed by Western blot as described [18] using specific primary antibodies: rabbit anti-TLR4, mouse anti-phospho-P38, rabbit anti-phospho-ERK1/2, rabbit anti-phospho-JNK1/2, rabbit anti-P38, rabbit anti-ERK1/2, mouse anti-JNK1/2, mouse anti-p-NF-*κ*B, rabbit anti-Bax, rabbit anti-Bcl2, and mouse anti-*β*-actin at 1 : 1000 dilution.

### 2.4. Measurement of Nitrite Production

The nitrite production was examined using Griess reagent (Sigma-Aldrich) as described previously [19].

### 2.5. Cell Survival Assay

Cell viability was measured using MTT assay (Sigma-Aldrich) as per manufacturer's guidelines.

### 2.6. Neuro2a-BV2 Microglia Coculture

Microglial mediated neurotoxicity was evaluated using neuro2a-BV2 microglia coculture assay. Briefly, neuro2a cells were plated and “differentiated” on coverslips as previously described [20]. BV2 microglia cells were plated simultaneously in 24-well culture plates and pretreated with LPS-Rs (5 *μ*g/mL) and then stimulated with LPS (1 *μ*g/mL) for 48 hrs. Then, the neuron-containing coverslips (cell-side down) were moved into the microglia-seeded well, allowing neurons and microglia to share the same culture media, but without direct cell-cell contact (using three equally spaced small paraffin feet). The coverslips were moved out and neuronal viability was assessed using MTT assay. Morphologic examination of neurons was done under inverted phase-contrast microscope (Olympus).

### 2.7. Immunofluorescence

Nuclear translocation of NF-*κ*B p65 was analysed by immunofluorescence assay. Briefly, BV2 cells were grown for 24 hrs and then treated as indicated followed by 1 *μ*g/mL LPS treatment for 60 min. After washing, cells were fixed with 1% paraformaldehyde for 5 min at room temperature and then permeabilized with PBST (PBS containing 0.2% Triton X-100). After blocking in the blocking buffer (2% BSA in PBST) for 2 hrs the cells were sequentially incubated with mouse anti-NF-*κ*B p65 antibody (Invitrogen) and secondary antibody anti-mouse IgG (Alexa Fluor 488). 4,6-Diamidino-2-phenylindole (DAPI 1 *µ*g/mL) was used to visualize nucleus and cells were examined under fluorescence microscope (Olympus).

### 2.8. Analysis of Phagocytic Activity

Measurement of intracellular* E. coli* DH5*α* was used to determine the phagocytosis ability as described [21]. Briefly, BV2 cells were pretreated with LPS-Rs or Cyto-D for 2 hrs followed by LPS treatment for 24 hrs. Cells were washed twice and infected with* E. coli* for 30 minutes and the extracellular bacteria were washed and killed with gentamicin (100 *µ*g/mL). The cells were washed, lysed, and plated overnight on Luria-Bertani agar and colony forming unit (CFU) was calculated. In parallel experiment, phagocytic activity was evaluated by fluorescence microscopy. In brief, for staining of* E. coli* DH5*α* with DAPI, the* E. coli* DH5*α* were incubated with DAPI (1 *µ*g/mL) for 5 min and extracellular DAPI was removed by 4 times washing at 12,000 g for 3 min at 25°C. Then microglial cells were infected with DAPI stained* E. coli* DH5*α* for 30 min followed by three washes with PBS and phagocytosis was evaluated by fluorescence microscopy at 40x magnification.

### 2.9. TLR4 Knockdown by siRNA Transfection

We examined the role of TLR4 in microglia-mediated neuroinflammation and neuronal cell death. Briefly, BV2 microglia were transfected under serum-free conditions with TLR4 siRNA or control siRNA (1 *µ*g/mL). TLR4 siRNA sequence (sense 5′-GGA ACU UGG AAA AGU UUG-3′) and control siRNA (sense 5′-GUG CAC AUG AGU GAG AU UU-3′) in pSilencer 4.1-CMV neo siRNA expression vector were used as described previously [22] and transfected using Lipofectamine 2000 (Invitrogen), according to the manufacturer's instructions. After overnight incubation the medium was replaced and the cultures were continued for 48 hrs before the assays were performed.

### 2.10. Splenocytes Functional Assays

The experiments of splenocytes from C57BL/6 mice were performed as per the guidelines and protocol approval by Committee for the Purpose of Control and Supervision of Experiments on Animals (CPCSEA). RBC depleted splenocytes were pretreated either with or without LPS-Rs followed by LPS treatment for 72 hrs [23]. The cells were then analysed for proliferation using MTT assay, Treg population using flow cytometry, and cytokines using ELISA. For RT-PCR the gene expressions were determined 48 hrs after the treatment. CD4^+^ T cells were isolated following negative selection using streptavidin-Imag beads. To confirm the immunosuppressive function of Treg cells, CD4^+^ T cells were treated with recombinant (r) IL-2 in presence or absence of rIL-10 and the supernatants were used for pretreatment of microglia followed by stimulation with LPS, and the inflammatory function of microglia was determined by measurement of TNF-*α* and NO.

### 2.11. Flow Cytometry

Mouse regulatory T cell staining kit was used for staining as per the manufacturer's instructions (eBioscience). All data were collected on a FACS Calibur (BD Biosciences). CD4^+^CD25^+^ cells were gated and the expression of Treg specific marker Foxp3 was determined using CellQuest software.

### 2.12. Cytokine ELISA

Cytokines in the culture supernatants were detected using ELISA for the indicated cytokines as per manufacturer's guideline (eBioscience).

### 2.13. Statistical Analyses

Each individual experiment was repeated a minimum of three times and the statistical significance of differences between groups was determined by one-way ANOVA followed by Tukey's post hoc multiple comparison tests. The data were expressed as mean ± SEM from three independent experiments. A statistical *p* value less than 0.05 (*p* ≤ 0.05) was considered significant (^*∗*^ *p* ≤ 0.05 versus untreated control and ^#^ *p* ≤ 0.05 versus cells treated with LPS).

## 3. Results

### 3.1. LPS-Rs Inhibits Microglia Activation and Inflammation

**Figure 1:**
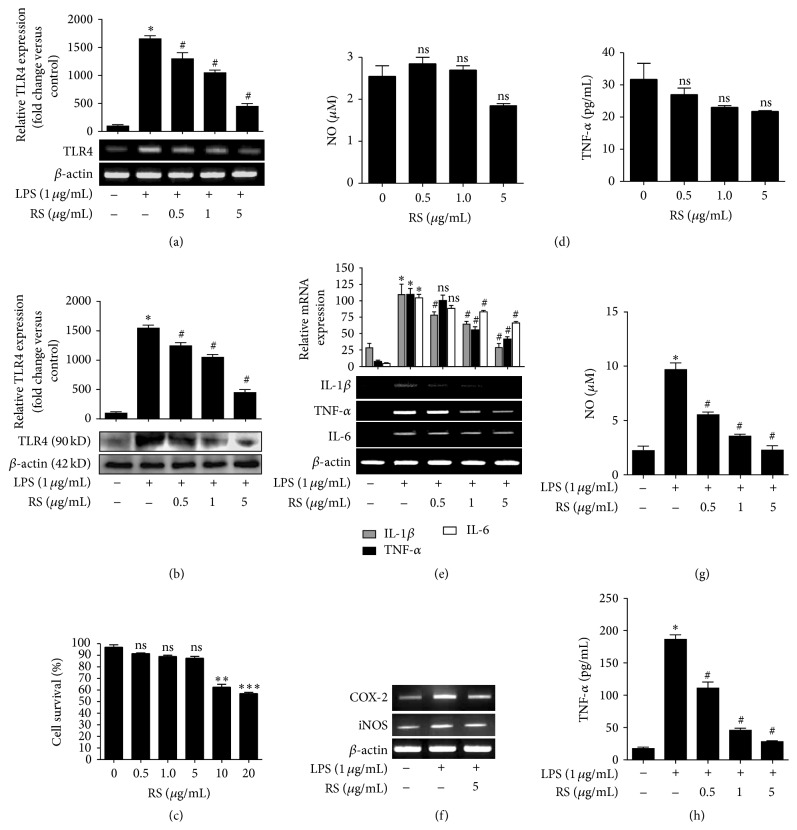
LPS-Rs inhibits microglia activation and inflammatory response. Microglial cells were pretreated with indicated concentration of LPS-Rs for 2 hrs before treatment of LPS for indicated time. Expression of TLR4 was assayed by semiquantitative RT-PCR (6 hrs) (a) and Western blot (b) (24 hrs). The cell viability was measured by MTT assay (c) and NO and TNF-*α* production was measured by Griess reagent and ELISA, respectively (d). The mRNA expressions of IL-1*β*, TNF-*α*, IL-6 (e), iNOS, and COX-2 (f) were examined by semiquantitative RT-PCR (6 hrs). Nitrite released into the culture medium was assayed using Griess reagent (g) and TNF-*α* in the culture supernatant was measured using ELISA (h). The error bars represent the mean ± SEM from three independent experiments (^*∗*^ *p* ≤ 0.05 versus control and ^#^ *p* ≤ 0.05 versus LPS). UN: untreated; LPS: lipopolysaccharide from* Escherichia coli* 055 : B5 (1 *µ*g/mL); RS: lipopolysaccharide from* Rhodobacter sphaeroides* (0.5–5 *µ*g/mL).

We investigated the effect of LPS-Rs mediated regulation of LPS induced microglia activation, expression of TLR4, and inflammatory cytokines. BV2 microglial cells were pretreated with LPS-Rs (0.5–5 *µ*g/mL) for 2 hrs followed by treatment with LPS (1 *µ*g/mL) and expression of TLR4 was analyzed by RT-PCR (6 hrs) (Figure 1(a)) and TLR4 Western blot (24 hrs) (Figure 1(b)). LPS-Rs markedly reduced TLR4 expression compared to the LPS. The concentrations of LPS-Rs used did not induce cytotoxicity and NO and TNF-*α* production (Figures 1(c) and 1(d)). LPS-Rs significantly decreased TLR4 induced cytokines* tnf-α*,* il-1β*, and* il-6* (Figure 1(e)) and inflammatory genes* inos* and* cox-2* (Figure 1(f)) accompanied with decreased NO and TNF-*α* production (Figures 1(g) and 1(h)). Microglial morphology data provides evidence that LPS-Rs ameliorate microglia activation as ramified morphology was regained (supplemental Figures S1A, S1B, and S1C). These data indicate that LPS-Rs exhibited a broad spectrum of inhibitory effects on microglia activation and production of inflammatory mediators.

### 3.2. LPS-Rs Negatively Regulates NF-*κ*B and MAPKs Signaling Pathways

**Figure 2:**
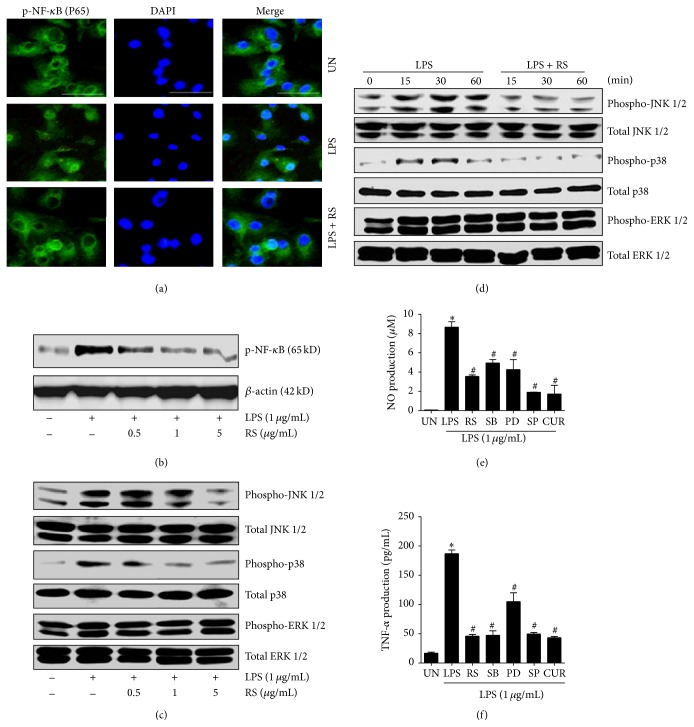
Effect of LPS-Rs on phosphorylation of NF-*κ*B and MAPKs and their indispensable role in LPS activated microglia. Microglia were pretreated with LPS-Rs followed by LPS for indicated time. Phosphorylation and translocation of p-65 NF-*κ*B were detected after 1 hr by immunofluorescence (a) (scale bars = 50 *μ*m) and Western blot (b). Phosphorylated and total protein expression of MAPKs was detected by Western blotting assay using specific antibodies (c, d). Production of TNF-*α* (e) and nitric oxide (f) into the culture medium was analysed after 24 hrs by ELISA and Griess reagent, respectively. The error bars represent the mean ± SEM from three independent experiments (^*∗*^ *p* ≤ 0.05 versus control and ^#^ *p* ≤ 0.05 versus LPS). UN: untreated, LPS: lipopolysaccharide from* Escherichia coli* 055 : B5 (1 *µ*g/mL), RS: lipopolysaccharide from* Rhodobacter sphaeroides* (0.5–5 *µ*g/mL), SB: SB202190 (10 *µ*M), PD: PD184352 (5 *µ*M), SP: SP600125 (10 *µ*M), and CUR: Curcumin (10 *µ*M).

NF-*κ*B phosphorylation and nuclear translocation are important events that trigger transcription of inflammatory genes [18, 24]. LPS-Rs significantly inhibited LPS induced phosphorylation and nuclear translocation of p-65 NF-*κ*B in a dose dependent manner (Figures 2(a) and 2(b) and supplemental Figure S2). NF-*κ*B luciferase reporter assay also confirms the same (supplemental Figure S3). MAPKs, playing an important role in the microglia activation and inflammation, are considered potential targets for treatment of neuroinflammatory diseases [18, 20, 25–27]. We examined the regulatory effects of LPS-Rs on MAPK pathways in dose and time dependent manner. LPS-Rs inhibits LPS-induced phosphorylation of JNK 1/2 and p38 MAPKs but left ERK 1/2 unaltered (Figures 2(c) and 2(d)). Consequent to the above observations, the essentiality of these pathways was determined using inhibitors and the NF-*κ*B and JNK1/2 were found to be indispensable for LPS induced NO and TNF-*α* secretion (Figures 2(e) and 2(f)). Collectively, the results suggest that LPS-Rs prevents neuroinflammation through negative regulation of NF-*κ*B and/or MAPKs.

### 3.3. LPS-Rs Prevents Microglia-Mediated Neuronal Insults

**Figure 3:**
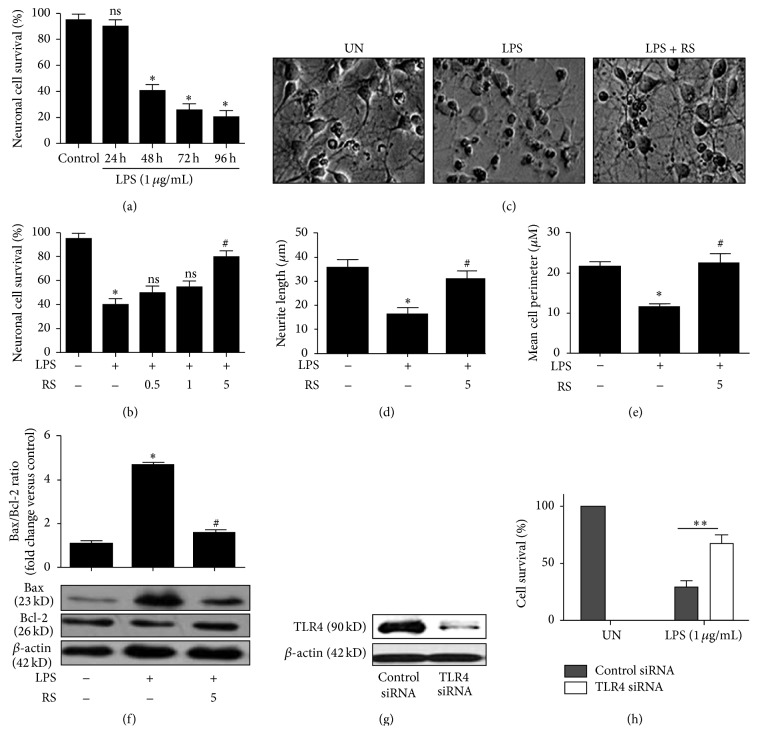
LPS-Rs ameliorates microglia-mediated neuronal insults. BV2 cells were differentially treated in absence and presence of LPS-Rs followed by LPS treatment as indicated. Later the differentiated neuro2a cells were cocultured with BV2 cells and neuronal viability was measured using MTT and morphological observation (b, c, d). Supernatants from conditioned microglial cells were used to treat differentiated neuro2a cells and the Bax : Bcl2 ratio was measured following Western blot (e). BV2 cells were transfected with control or TLR4 siRNA and TLR4 silencing was assayed by Western blot (f). TLR4 silencing suppressed LPS induced neuronal cell death (g). The error bars represent the mean ± SEM from three independent experiments (^*∗*^ *p* ≤ 0.05 versus control and ^#^ *p* ≤ 0.05 versus LPS). UN: untreated; LPS: lipopolysaccharide from* Escherichia coli* 055 : B5 (1 *µ*g/mL); RS: lipopolysaccharide from* Rhodobacter sphaeroides* (0.5–5 *µ*g/mL).

It is well documented that activated microglia significantly contributes neuronal apoptosis and subsequent clearance [10, 28]. Aberrant activation of microglia initiates a series of inflammatory cascades that leads to deregulated apoptosis. In neuro2a-BV2 microglia coculture, LPS activated microglia induces neuronal cell death which was inhibited significantly by LPS-Rs (Figures 3(a) and 3(b)). TLR4 agonist, LPS, and its antagonist, LPS-Rs, did not induce neuronal cell death in the differentiated neuro2a cells (data not shown). The observation affirms the essential role of microglia in mediating neuronal cell death. LPS activated microglia-induced abnormal neuronal morphology together with fragmentation of neurites and shrunken cell bodies, which were significantly inhibited by LPS-Rs affirming its neuroprotective functions (Figure 3(c)) including neurite length and cell perimeter (Figures 3(d) and 3(e)). The effect of LPS-Rs on the intrinsic apoptotic pathway was investigated by determination of the Bax : Bcl-2 ratio [29]. Exposure of differentiated neuro2a cells to supernatant from differentially treated microglia accounted for threefold decrease in the ratio that was significantly high during LPS treatment (Figure 3(f)) suggesting inhibition of apoptotic cell death. To examine and confirm the functional role of TLR4 in microglia-mediated neurotoxicity, the expression was silenced using siRNA and expression level confirms silencing (Figure 3(g)). TLR4 silencing attributes to decreased production of inflammatory mediators TNF-*α* and NO after LPS treatment (supplemental Figure S4). Exposure of differentiated neuro2a cells to the supernatant from control siRNA and TLR4 siRNA transfected LPS stimulated microglia showed increased neuronal cell death in the control while the effect was significantly inhibited in TLR4 siRNA transfectants. Cell viability: TLR4 siRNA + LPS: 75%  ± 3.5, control siRNA + LPS: 30%  ± 3, *p* < 0.005 (Figure 3(h)). To extrapolate our findings with an* in vitro* model of AD, we confirmed the potential role of LPS-Rs in rescuing *β* amyloid induced neuroinflammation and neuronal loss (supplemental Figure S5). Collectively, LPS-Rs and/or TLR4 silencing inhibits LPS induced microglia-mediated neuronal apoptosis via inhibition of inflammation in the neuro2a-BV2 microglia coculture system.

### 3.4. LPS-Rs Ameliorates Phagocytic Activity of LPS Activated Microglia

**Figure 4:**
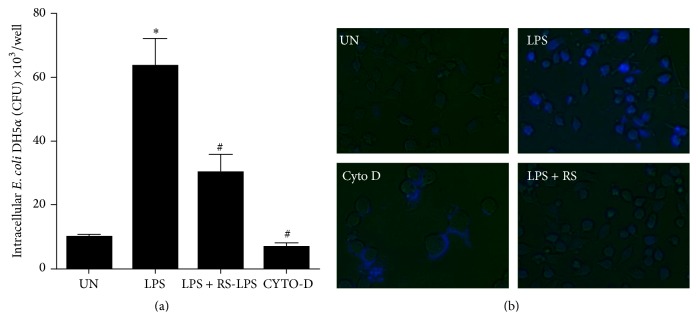
LPS-Rs diminished microglial phagocytic activity. Microglia phagocytic activity was measured by bacterial colony forming unit (CFU) count (a). BV2 microglia were pretreated with LPS-Rs (5 *μ*g/mL) or Cytochalasin D (10 *µ*M) for 2 hrs before LPS (1 *μ*g/mL) treatment for 24 hrs. The cells were then infected with DAPI stained* E. coli* DH5*α*. After 30 min gentamicin (200 *µ*g/mL) was added for 1 hr to kill extracellular bacteria and intracellular bacterial load was assayed using fluorescence microscopy at 40x magnification (b). The error bars represent the mean ± SEM from three independent experiments (^*∗*^ *p* ≤ 0.05 versus control and ^#^ *p* ≤ 0.05 versus LPS). UN: untreated; LPS: lipopolysaccharide from* Escherichia coli* 055 : B5 (1 *µ*g/mL); RS: lipopolysaccharide from* Rhodobacter sphaeroides* (0.5–5 *µ*g/mL); Cyto D: Cytochalasin D.

Microglia plays crucial role in phagocytosis of apoptotic or dead neurons, studies have demonstrated that the mechanisms involve either eat-me signal on the target cell or coreceptor on the microglia cell surface. Modulation of phagocytosis is important to prevent neurodegenerative processes [30]; hence, we examined whether phagocytic activity of microglia can be regulated by LPS-Rs. Unstimulated cells ingested low number of bacteria (10 × 10^3^ CFU/mL) while LPS activated microglia ingested highest number of bacteria (80 × 10^3^ CFU/mL; *p* < 0.0002). Microglia pretreated with LPS-Rs phagocytosed significantly less number of bacteria (25 × 10^3^ CFU/mL; *p* < 0.0002) and the phagocytic inhibitor Cytochalasin D pretreatment acted as a positive control with least number of ingested bacteria (8 × 10^3^ CFU/mL; *p* < 0.0002) (Figure 4(a)). Microscopic analysis of labeled bacteria confirms the inhibitory potential of LPS-Rs for phagocytosis which is otherwise aberrantly activated upon TLR4 activation (Figure 4(b)). The data indicates that LPS-Rs inhibits LPS induced microglial phagocytic activity that may serve as a key and sole strategy to prevent neurodegeneration [30].

### 3.5. LPS-Rs Inhibits Costimulatory and Leukocyte Trafficking Molecules

**Figure 5:**
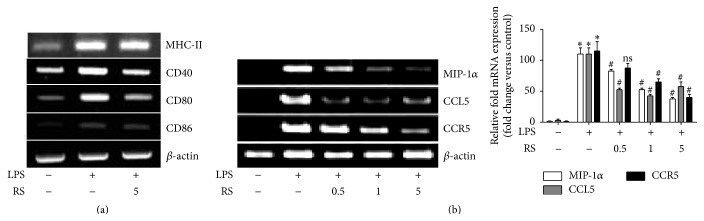
LPS-Rs inhibit costimulatory molecules and leukocyte trafficking factors required for T cell interaction and infiltration. BV2 microglia were pretreated with LPS-Rs for 2 hrs followed by LPS treatment for 6 hrs and the mRNA expression of macrophage activation markers: MHC-II, CD40, CD80, and CD86 (a) and chemokines and chemokine receptors (MIP-1*α*, CCL5, and CCR5) (b) were detected by RT-PCR. The error bars represent the mean ± SEM from three independent experiments (^*∗*^ *p* ≤ 0.05 versus control and ^#^ *p* ≤ 0.05 versus LPS). UN: untreated; LPS: lipopolysaccharide from* Escherichia coli* 055 : B5 (1 *µ*g/mL); RS: lipopolysaccharide from* Rhodobacter sphaeroides* (0.5–5 *µ*g/mL).

Appropriate antigen processing and presentation are the key governing factors that orchestrate the T cell response. During the neuroinflammatory burden the T cells are biased to be activated, aggravating the problem. The governing factors deciding the fate of T cell differentiation are expression of costimulatory molecules and chemokine receptors accompanied with release of cytokines: the releases of chemoattractants, chemokines, and guide T cells infiltration to the site of injury/infection. LPS induced elevated levels of the key costimulatory molecules and activation markers* cd80*,* cd86*, and* cd40* were negatively regulated upon pretreatment with LPS-Rs (Figure 5(a)). LPS-Rs however did not show significant modulation in the expression of* mhc-ii* gene. Researchers have highlighted the fact that TLR4 is indispensable for leukocyte recruitment into brain in response to LPS [31] and upregulated expression of CCR5 in neurological diseases is often immunolocalized in microglia [32]. We report similar observation that LPS stimulation results in significantly elevated expression of* ccr5* which was downregulated by LPS-Rs. The chemokines, including* mip-1α* and* ccl5*, are the major activator and chemoattractants for monocytes and T cells [8] and were significantly inhibited by LPS-Rs (Figure 5(b)). Our finding indicates that LPS-Rs mediate inhibition of gene expression of these costimulatory molecules, chemokines, and chemokines receptor which may govern microglia-T cell interaction and T cell infiltration into the brain.

### 3.6. LPS-Rs Inhibit Lymphocyte Proliferation and Induce Treg Population

**Figure 6:**
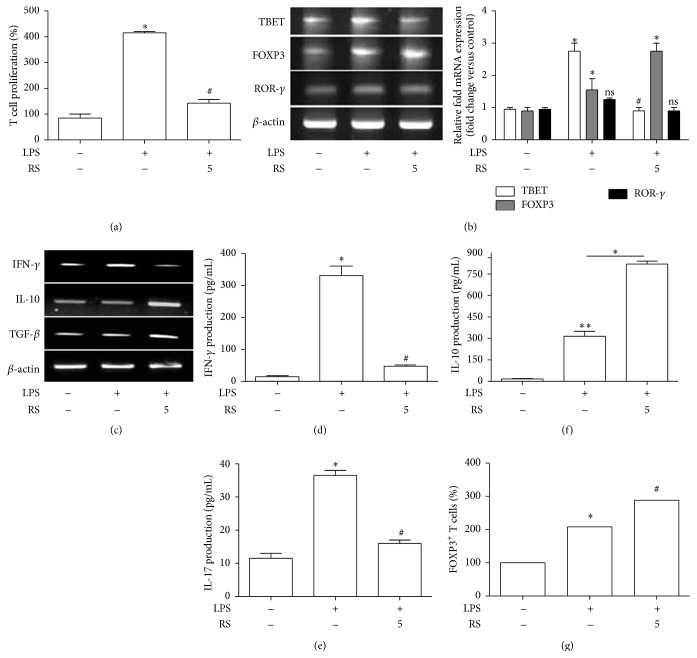
LPS-Rs skews T cell response with induction of Tregs cells. Splenocytes were treated with or without LPS-Rs followed by LPS treatment and cell proliferation was assayed by MTT assay after 72 hrs (a). T helper associated transcription factors: TBET, FOXP3, and ROR-*γ* (b) and cytokines IFN-*γ*, IL-10, and TGF-*β* (c) were assayed by RT-PCR after 48 hrs. The secretary IFN-*γ*, IL-17, and IL-10 from culture supernatant were analysed by ELISA after 72 hrs (d, e, and f). The differentially treated splenocytes after 72 hrs were fixed, surface stained with FITC-CD4 mAb and APC-CD25 mAb followed by permeabilization and intracellular staining with PE-Foxp3 mAb, and analyzed by flow cytometry. The CD4^+^CD25^+^ cells were gated and Foxp3 expression was determined and expressed as percentage change (g). The error bars represent the mean ± SEM from three independent experiments (^*∗*^ *p* ≤ 0.05 versus control and ^#^ *p* ≤ 0.05 versus LPS). UN: untreated; LPS: lipopolysaccharide from* Escherichia coli* 055 : B5 (1 *µ*g/mL); RS: lipopolysaccharide from* Rhodobacter sphaeroides* (0.5–5 *µ*g/mL).

The activation state (activated or tolerogenic) of the antigen presenting cells and subsequent events dictate adaptive immune functions. Tolerogenic APCs can be potential target to design and direct effective strategy to regulate inflammatory and pathogenic T cells. We have tried to understand the activating/tolerogenic effect of LPS-Rs in splenocyte cultures that may regulate the T cell outcome. LPS-Rs resulted in approximately 3-fold lesser proliferation as compared to the cells activated by LPS (Figure 6(a)). To further correlate reduced proliferation with functional markers for the T cells, the expressions of T helper associated transcription factors and secretary cytokines were analysed. LPS remarkably induced transcription factors and cytokines for Th1 (*tbet and IFN-γ*) and Th17 (*ror-γ* and IL-17) cells. Interestingly, pretreatment with LPS-Rs demonstrated that T cells differentiated to Treg lineage as evidenced by its signature transcription factor and cytokines (*foxp3*,* tgf-β*, and IL-10) (Figures 6(b)–6(f)). To confirm the phenotype, the cells from parallel experiment were analysed for Treg surface marker (Foxp3^+^) using flow cytometry. LPS-Rs pretreatment significantly enhanced Foxp3 expression (40%) on CD4^+^CD25^+^ gated cells as compared to LPS stimulation alone (Figure 6(g)). The generation of T regulatory cells during LPS-Rs pretreatment can be a possible reason for reduced proliferation [33, 34]. Taken together, the data suggest that LPS treatment directs Th1/Th17 differentiation while pretreatment with LPS-Rs confers induction of Tregs cells.

### 3.7. Treg Cells Confer Tolerogenic Potential to Microglia

**Figure 7:**
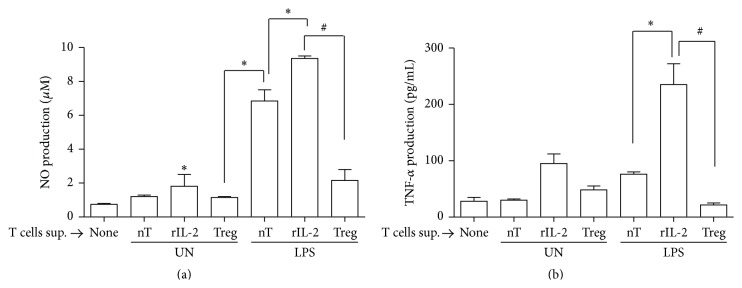
Treg cells confer tolerogenic potential to microglia. CD4^+^ T cells were treated with recombinant IL-2 in presence or absence of IL-10 for 72 hrs and the supernatants were used for pretreatment of microglia followed by stimulation with LPS; production of nitric oxide (a) and TNF-*α* (b) into the culture medium was analysed by Griess reagent and ELISA, respectively. The error bars represent the mean ± SEM from three independent experiments (^*∗*^ *p* ≤ 0.0001 versus control and ^#^ *p* ≤ 0.0007 versus LPS). T cells sup.: supernatant from T cells; UT: untreated; rIL-2: recombinant IL-2; rIL-10: recombinant IL-10.

T regulatory cells are known to potent suppressors of the adaptive immune system and also modulate innate immune cells through induction of alternative activation of macrophages [35]. Studies have shown that Foxp3^+^CD4^+^ Treg cells play crucial role in maintenance of immunological homeostasis and tolerance in T lymphocytes and macrophages [36]. Several studies have shown that alternative (anti-inflammatory/tolerogenic) activation of microglia is a beneficial response to CNS injury [36, 37]. Cellular factors influencing microglial fate include CNS infiltrating T cells amongst others. Infiltrating CD4^+^ T-cells participate and influence microglial activation and consequent neuronal damage. Microglia may acquire inflammatory neurotoxic phenotype or immunosuppressive neurosupportive phenotype [15, 38]. To examine whether immunosuppressive and/or tolerogenic potential of Treg cells manipulate activation state of microglia, we used anti-CD3/CD28 stimulated CD4^+^ T cells and treated them in presence or absence of rIL-10 along with rIL-2. The cells treated with rIL-2 in absence of rIL-10 showed enhanced proliferation and absence of Treg phenotype while the cell treated with r-IL-2 in presence of rIL-10 demonstrated reduced T cell proliferation and expressed Treg marker (FOXP3^+^) (data not shown). The supernatants from the experiment were collected and were used to pretreat microglia followed by LPS stimulation. The supernatant from rIL-10 induced Treg cells significantly decreased secretion of NO and TNF-*α* from microglia as compared to control (Figures 7(a) and 7(b)). The results indicate that rIL-10 induced Treg cells negatively regulate inflammatory function of microglia and render them to gain immunosuppressive or tolerogenic functions.

## 4. Discussion

In brain pathology, inflammation causes bystander injury that is typically irreversible followed by sustained neuronal and cognitive function loss. The CNS exhibits TLRs, including TLR4, has predominant expression on microglia, and initiates typical response to CNS infection or injury [10, 12, 28, 39]. However, in some cases activation of microglia contributes to neurodegeneration by releasing inflammatory and cytotoxic factors, including nitric oxide and TNF-*α* as in AD and PD [18, 40].

Reports highlight the fact that upregulated TLR4 initiates inflammation and increases phagocytic activity of microglia followed by neuronal loss in AD and PD [10, 12, 39]. TLR4 deficiency protects mice against neurodegeneration and showed increased survival of neurons confirming the involvement of the receptor [13]. Mutation in the receptor decreases microglial activation and preserves cognitive functions in mouse model of AD [14]. Thus, TLR4 activation is the major culprit for microglia-mediated neuronal loss as demonstrated by many leading groups. In the same context, our study using a TLR4 antagonist, LPS-Rs, reveals that LPS-Rs markedly inhibits TLR4 expression with substantial and subsequent decrease of inflammatory mediators including IL-1*β*, TNF-*α*, IL-6, iNOS, and COX-2. Our findings are supported by studies that highlighted the deficiency and mutation in TLR4 benefits in preserving cognitive functions and better neuronal well-being [13, 14].

Elevated activation of NF-*κ*B and MAPKs is directly involved in pathogenic events of AD and PD [18, 20, 24–27, 41]. NF-*κ*B is an important factor in regulation of microglia-mediated neuroinflammatory response [42]. NF-*κ*B exists in the cytosol as a dimer of p-65 NF-*κ*B and I*κ*B, an associated inhibitory protein. Upon activation the phosphorylated p-65 NF-*κ*B translocates to the nucleus inducing a cascade of inflammatory genes [42, 43]. In addition to NF-*κ*B, MAPKs, playing an important role in the microglia activation and secretion of inflammatory mediators, are considered potential targets for treatment of neuroinflammatory diseases [26, 27]. In this regard, our findings indicate that LPS-Rs significantly blocks LPS induced phosphorylation of MAPKs and NF-*κ*B p65. Thus, LPS-Rs can be exploited as possible mechanisms for better treatment regimes.

Activated microglia through inflammation influences neuronal apoptosis and progression of neurodegenerative diseases [6, 12, 28, 44]. Using neuro2a-BV2 microglia coculture we report that LPS induced microglial neurotoxicity was significantly inhibited by LPS-Rs and the protective effects might be due to its inhibitory action on aberrant neurotoxic activation. Pretreatment with LPS-Rs significantly reduced the Bax : Bcl2 ratio in “differentiated” neuro2a cells exposed to supernatant from LPS activated microglia resulting in better neuronal survival. To confirm the role of TLR4 in microglial activation and neurodegenerative processes, we silenced the expression of TLR4 using siRNA. Silencing of TLR4 was sufficient to confirm its role in inflammatory neuronal loss and was confirmed with *β* amyloid as well. Our findings are consistent with the evidence that reports neuroprotective effects of TLR4 antagonism in spinal cord and mouse model of neurodegeneration [45]. We have exemplified with a strong background that LPS-Rs is a potential candidate to recover TLR4 mediated neuronal damage.

Damaged, dead, or dying neurons are phagocytosed by microglia to maintain homeostasis in the CNS but aberrantly activated microglia fails to discriminate between live and damaged/dead neurons, resulting in phagocytic removal of live neurons, contributing to significant neurodegeneration [8, 18, 44]. Recent study has shown that loss of neurons during inflammation, executed by microglial phagocytosis and its inhibition, is sufficient to prevent inflammatory neuronal death [30]. In this regard, we have explored the potential of LPS-Rs as phagocytic inhibitor and demonstrate that it significantly blocks phagocytosis of* E. coli* DH5*α* in LPS activated microglia, as analysed by fluorescent microscopy and CFU of ingested* E. coli* DH5*α*. The results provide a novel mechanism of LPS-Rs antagonism which has never been explored earlier and may provide excellent therapeutic strategy.

Furthermore, CNS infection and/or injury initiates leukocyte trafficking in the brain that has earlier been reported to worsen disease outcome. However, there are still discrepancies in the reports [15, 31, 46, 47]. The CD4^+^CD25^+^ regulatory T cells have neuroprotective activities through regulation of microglia activation and T cell functions by secreting immunosuppressive cytokines [48, 49]. In this regard our data supports the neuroprotective potential of CD4^+^CD25^+^ Treg cells. We demonstrate that TLR4 antagonism by LPS-Rs not only rescues microglia-mediated inflammatory events but also results in Treg induction, and the findings are also supported by other studies [50]. Naturally occurring Treg cells express Foxp3, the key regulator of Treg cell development and function [51]; studies using Treg suggest suppressive function on T cell immune responses [34, 52]. Therefore, T reg cells can mediate their actions by attenuating inflammatory responses, thus ameliorating neuronal degeneration [53], yet the exact mechanism of their actions in the injured CNS is poorly understood. To provide a better understanding in this context, using the conditioned cytokines we have shown that Tregs can transfer their tolerogenic functions to microglia as evidenced by decreased TNF-*α* and NO production. Our data strongly recommend that TLR4 antagonism can be a strategy providing neuroprotection through regulation of microglia as well as the T cells.

## 5. Conclusion

Conclusively, the study reports that LPS-Rs prevents TLR4 induced neuroinflammation and microglia activation via negative regulation of NF-*κ*B and MAPKs signaling pathways conferring decreased neuronal apoptosis and subsequent phagocytosis by the microglia. LPS-Rs through TLR4 antagonism not only regulates microglial function but also may serve to induce Treg cells that support the tolerogenic microenvironment and further strategies may be designed and implemented not only for CNS diseases but also for other disease regimens.

## Supplementary Material

Table 1. List of primers used in the study.
Figure S1. TLR4 antagonism inhibits LPS induced microglia activation.
Figure S2. Rs-LPS blocks phosphorylation and translocation of NF-κB in LPS activated BV-2 microglia
Figure S3. NF-κB luciferase reporter assay.
Figure S4. TLR4 silencing attributes to decreased production of inflammatory mediators like TNF-α and NO after LPS treatment.
Figure S5. TLR4 antagonism confers neuroprotection against Aβ induced inflammatory damages.

## Abbreviations

AD: Alzheimer's disease
CCL5: Chemokine (C-C motif) ligand 5
CCR5: C-C chemokine receptor type 5
CD25^+^: Cluster of differentiation 25 positive
CD4^+^: Cluster of differentiation 4 positive
CFU: Colony forming unit
COX-2: Cyclooxygenase-2
CUR: Curcumin
Cyto D: Cytochalasin D
ERK: Extracellular-signal-regulated kinases
FOXP3: Forkhead box P3
IFN-*γ*: Interferon-*γ*
IL-10: Interleukin-10
IL-17: Interleukin-17
IL-1*β*: Interleukin-1*β*
IL-6: Interleukin-6
iNOS: Inducible nitric oxide synthase
JNK: c-Jun N-terminal kinases
LPS: Lipopolysaccharide from* Escherichia coli* 055 : B5
LPS-Rs or RS: Lipopolysaccharide from* Rhodobacter sphaeroides*
MAPKs: Mitogen-activated protein kinases
MHC-II: Major histocompatibility complex class II
MIP: Macrophage Inflammatory Protein
NF-*κ*B: Nuclear factor kappa-enhancer of activated B cells
NO: Nitric oxide
p38: p38 mitogen-activated protein kinases
PD: PD184352
rIL: Recombinant interleukin
ROR-*γ*: RAR-related orphan receptor gamma
SB: SB202190
siRNA: Small interfering RNA
SP: SP600125
TGF-*β*: Transforming growth factor beta
Th: T helper
TLR4: Toll-like receptor 4
TNF-*α*: Tumor necrosis factor *α*
Treg cells: Regulatory T cells.
