# Supplementary material for: Lipopolysaccharide from Rhodobacter sphaeroides Attenuates Microglia-Mediated Inflammation and Phagocytosis and Directs Regulatory T Cell Response
Source: Int J Inflam. 2015 Sep 17;2015:361326. doi: 10.1155/2015/361326 (PMC4589630; doi:10.1155/2015/361326)
Supplement: Supplementary file 1 — Table 1. List of primers used in the study. Figure S1. TLR4 antagonism inhibits LPS induced microglia activation. Figure S2. Rs-LPS blocks phosphorylation and translocation of NF-κB in LPS activated BV-2 microglia Figure S3. NF-κB luciferase reporter assay. Figure S4. TLR4 silencing attributes to decreased production of inflammatory mediators like TNF-α and NO after LPS treatment. Figure S5. TLR4 antagonism confers neuroprotection against Aβ induced inflammatory damages. [file 361326.f1.zip › 361326.f1/SuplementaryMatreial.docx]

Supplemental Data

**Table1. List of primers used in the study.**

| **GENE** | **SEQUENCE** |
| --- | --- |
| TLR4 | FORWARD: ACTTGCCTTCAAAACCTGGC  REVERSE: GCAGAAACATTCGCCAAGCA |
| IL-1β | FORWARD: AGGAGAACCAAGCAACGACA  REVERSE: AGCTCCTTAACATGCCCTGG |
| IL-6 | FORWARD: AGTTGCCTTCTTGGGACTGA  REVERSE: TCCACGATTTCCCAGAGAAC |
| TNF-α | FORWARD: CATCCATTCTCTACCCAGCC  REVERSE: TCCAGCCTCATTCTGAGACA |
| iNOS | FORWARD: ACATGCTACTGGAGGTGGGT  REVERSE: GACTCTGAGGCTGTGTGGTG |
| COX2 | FORWARD: TGGGGGAAGAAATGTGCCAA  REVERSE: TGGCTGTTTTGGTAGGCTGT |
| β -actin | FORWARD: TCGAAGTCTAGAGCAACATAGCA  REVERSE: TCGAAGTCTAGAGCAACATAGCA |
| IFN-γ | FORWARD: GTGGAGCTTTGACGAGCACT  REVERSE:ATTCCCAGCATACGACAGGGT |
| MHC-II | FORWARD: GAGATCCTGGAGCGAACG  AGATGTTGTGGTGGTTGAG |
| CD80 | FORWARD: CTGTGACCATAAGAACTTGGAGG  REVERSE:AGAGAAACACCCCGAAAATGG |
| CD86 | FORWARD: TCAATGGGACTGCATATCTGCC  REVERSE:GCCAAAATACTACCAGCTCACT |
| IL-10 | FORWARD: GCCAAGCCTTATCGGAAATG  REVERSE:AAATCACTCTTCACCTGCTCC |
| CD40 | FORWARD: CTGTGACCATAAGAACTTGGAGG  REVERSE:AGAGAAACACCCCGAAAATGG |
| MIP-1α | FORWARD: CTGTGCTGATCCCAGTGAATC  REVERSE:TCAGTTCAGTTCCAGGTCATACA |
| CCL5 | FORWARD: TTTGCCTACCTCTCCCTCG  REVERSE:CGACTGCAAGATTGGAGCACT |
| CCR5 | FORWARD: ATGGATTTTCAAGGGTCAGTTCC  REVERSE:CTGAGCCGCAATTTGTTTCAC |
| TBET | FORWARD: TTCAACCAGCACCAGACAG  REVERSE:AGACCACATCCACAAACATCC |
| FOXP3 | FORWARD: AAGTACCACAATATGCGACCC  REVERSE:TCTGAAGTAGGCGAACATGC |
| ROR-γ | FORWARD: TTTCTGAGGATGAGATTGCCC  REVERSE:TTGTCGATGAGTCTTGCAGAG |

**S1. TLR4 antagonism inhibits LPS induced microglia activation. (A)**BV2 microglia were pre-treated with LPS-Rs for 2hrs followed by treatment with 1 μg/ml of LPS at 37°C for 24 hrs and stained with anti-β-actin (green), microglia morphology was observed under fluorescence microscope at 40x magnification. (B and C) LPS induced morphological alterations, mean cell perimeter and area in microglia cells were prevented by LPS-Rs. Morphometric parameters were measured using Image J software 120 cells from each group were analyzed from three independent experiments.


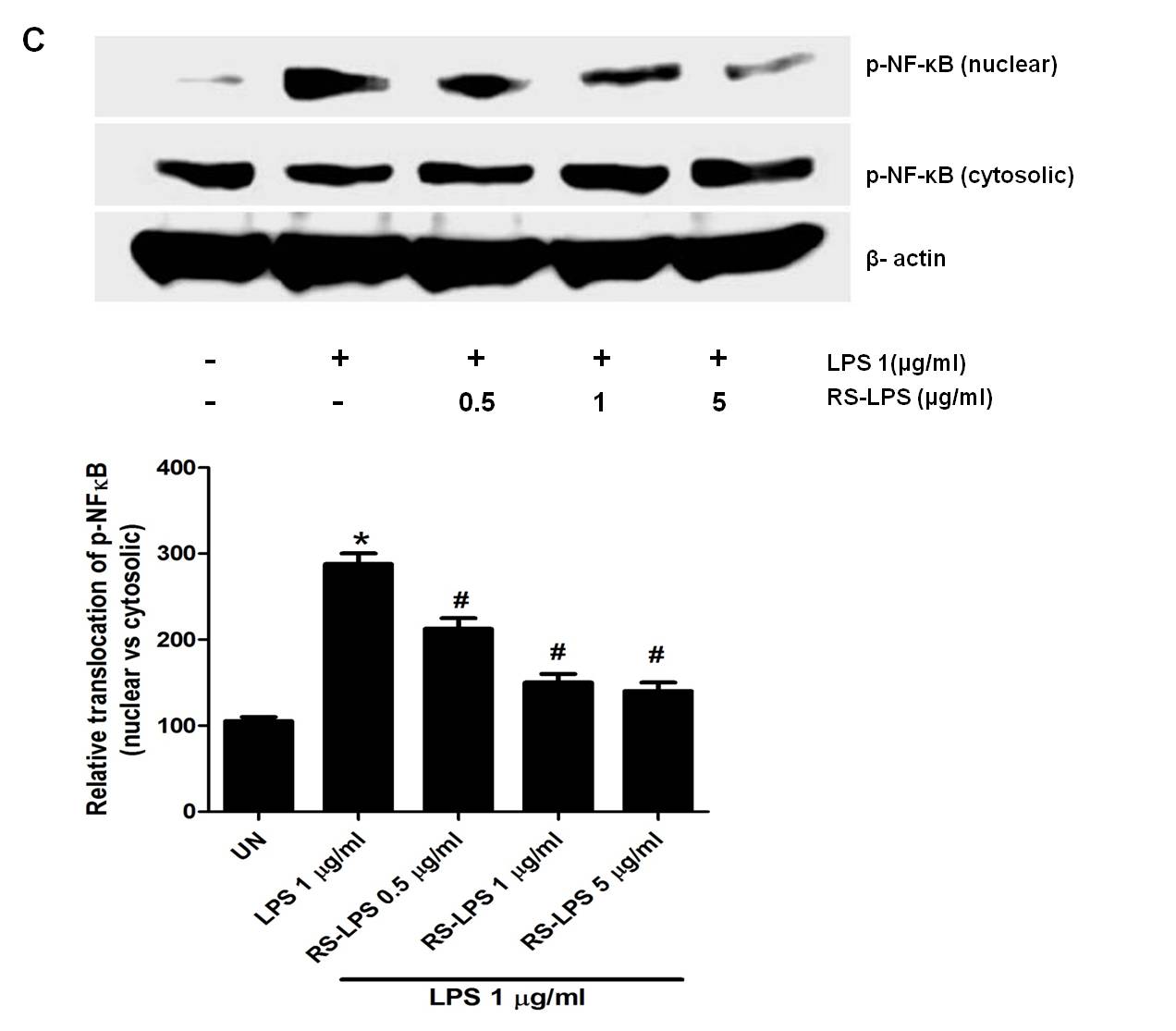


Figure S2. Rs-LPS blocks phosphorylation and translocation of NF-κB in LPS activated BV-2 microglia: BV2 microglia were pre-treated with different concentrations of RS-LPS for 2hrs followed by treatment with 1 μg/ml of LPS at 37°C for 60 min. Nuclear and cytosolic fraction were prepared and then phosphorylation and translocation of p65 NF-κB were detected by Western blotting assay using specific antibodies. Relative nuclear translocation of phospho-p65 NF-κB nuclear vs. cytosolic were expressed. The data are the mean ± SE (n = 3). *p < 0.05 vs. untreated control, #p<0.05 vs. cells treated with LPS.


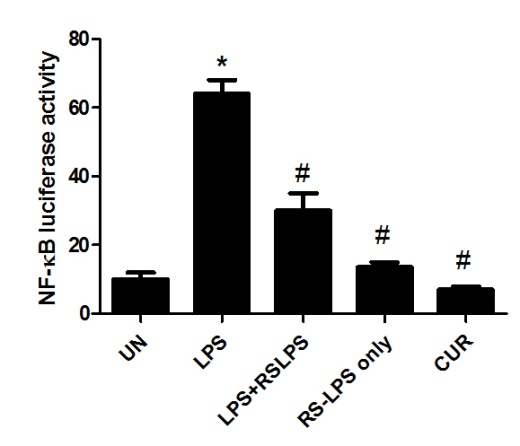


**Figure S3. NF-κB luciferase reporter assay:** BV2 cells were transfected with NF-κB promoter-luciferase reporter constructs were treated with or without 1µg/ml of LPS in the absence or presence of 5 µg/ml LPS-Rs for 12 hrs. After the treatment, cells were lysed and firefly luciferase activity was determined and normalized to renilla luciferase activity (C). The error bars represent the mean $\pm$SEM from three independent experiments. **P* ≤ 0.05 vs. untreated control, #P ≤ 0.05 vs. cells treated with LPS.

**Figure S4. TLR4 silencing attributes to decreased production of inflammatory mediators like TNF-α and NO after LPS treatment**. BV2 cells were transfected with control or TLR4 siRNA and stimulated with LPS for 24 hrs production of TNF-α (A) and nitric oxide (B) in to the culture medium was analysed by ELISA and Griess reagent, respectively.


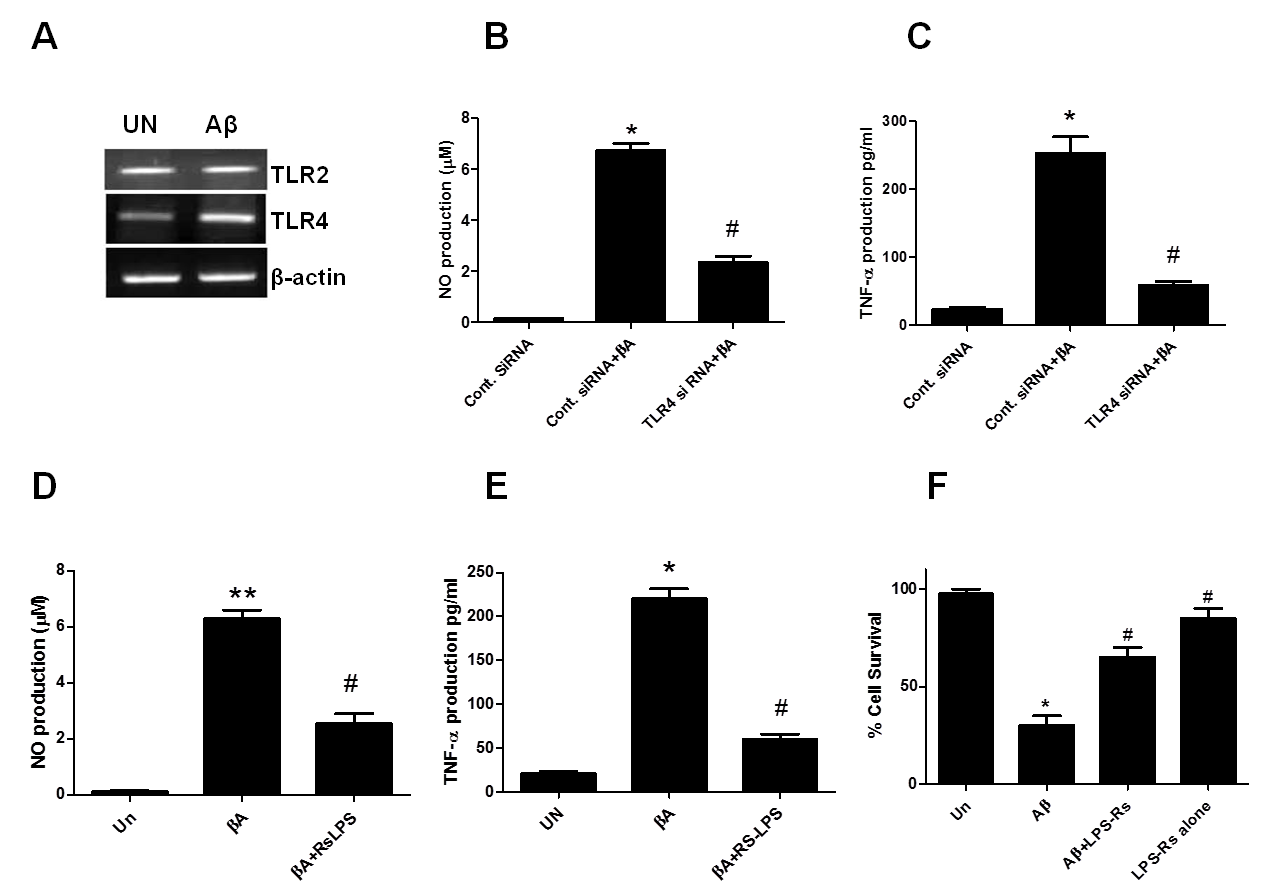


**Figure S5. TLR4 antagonism confers neuroprotection against Aβ induced inflammatory damages:** [A] BV2 microglial cells were stimulated with or without Aβ for 6 hrs. The mRNA expression of TLR2, TLR4 and β-actin was detected by RT-PCR. [B,C,D and E ] TLR4 silencing suppressed the increase of nitric oxide and TNF-α production in Aβ stimulated BV2 cells as assayed by Griess reagent and ELISA, respectively. [F] Differentiated neuro2a cells were exposed to supernatants from Aβ activated microglia pre-treated with or without LPS-Rs and neuronal cell viability was examined by MTT assay. The error bars represent the mean $\pm$SEM from three independent experiments. **P* ≤ 0.05.

**Supplemental experimental procedures**

**NF-κB nuclear translocation assay**

The NF-κB nuclear translocation was assayed as described previously by Sun et al., 2010 with few modifications. In brief, cells were washed with ice-cold PBS (pH 7.4) and then lysed for 15 min on ice in buffer A (10 mM HEPES buffer, pH 7.9, containing 0.1 mM EDTA, 10 mM KCl, 0.4% (v/v) NP-40, 0.5 mM dithiothreitol (DTT), and 1 mM phenylmethylsulfonyl fluoride (PMSF) followed by vigorous vortex for 15 seconds. Lysates were centrifuged at 6,000×g for 10 min. The resulting supernatants were collected as cytosolic fractions. The pellets were resuspended in buffer B (20 mM HEPES buffer, pH 7.9, 400 mM NaCl, 1 mM EDTA, 1 mM DTT and 1 mM PMSF) and incubated for 20 min on ice, then centrifuged at 14,000 ×g for 5 min. The resulting supernatants were used as nuclear extracts and translocation of phospho-p65 NF-κB was examined by western blot.

**Supplemental references**

Sun, Z., et al., 2010. Activation of PI3K/Akt/IKK-alpha/NF-kappaB signaling pathway is required for the apoptosis-evasion in human salivary adenoid cystic carcinoma: its inhibition by quercetin. Apoptosis. 15(7):850-63.
